# Synergistic Protective Effects of Oleaster Fruit and *Sophora japonica* L. Fruit Extracts Against IL-1β-Induced Inflammation in Human Chondrocytes

**DOI:** 10.3390/foods14173099

**Published:** 2025-09-04

**Authors:** Hana Lee, Jinyeong Lim, Myeonghwan Oh, Junsoo Lee

**Affiliations:** 1 Department of Food Science and Biotechnology, Chungbuk National University, Cheongju 28644, Chungbuk, Republic of Korea; dlgksk0514@naver.com; 2 Life Science Research Institute, NOVAWells Co., Ltd., Cheongju 28126, Chungbuk, Republic of Korea; jinyglim@novarex.co.kr

**Keywords:** oleaster, *Sophora japonica* L., synergy, osteoarthritis, inflammation, human chondrocytes

## Abstract

Osteoarthritis (OA) is a degenerative joint disease characterized by chronic inflammation and progressive cartilage breakdown. This study investigated the synergistic protective effects of oleaster fruit extract (OE) and *Sophora japonica* L. fruit extract (SJE) against interleukin-1β (IL-1β) -induced inflammation in human chondrocytes. OE and SJE were tested individually and in combination at various ratios (3:1, 2:1, 1:1, 1:2, and 1:3). Among the tested mixtures, the 3:1 OE:SJE ratio exhibited the most pronounced synergistic effect. When used individually, the concentrations required to achieve 90% cell viability were 313.6 µg/mL for OE and 4135.8 µg/mL for SJE. In contrast, the same viability level was achieved with a combined treatment of 26.4 µg/mL OE and 8.8 µg/mL SJE, yielding a combination index of 0.25, indicative of strong synergy. The 3:1 OE:SJE combination also significantly suppressed inflammatory mediators, including nitric oxide, interleukin-6, and tumor necrosis factor-α. This treatment also led to the downregulation of matrix-degrading enzymes, such as matrix metalloproteinase (MMP)-9 and MMP-13, and it promoted the preservation of hyaluronan, a key extracellular matrix component. These findings suggest that the 3:1 OE:SJE combination exerts a synergistic protective effect by modulating both inflammatory and catabolic pathways and may represent a promising therapeutic strategy for OA prevention or treatment.

## 1. Introduction

Osteoarthritis (OA) is the most prevalent chronic joint disorder worldwide, and its incidence continues to rise with aging populations and increasing obesity rates [1]. Clinically, OA is characterized by the progressive erosion of articular cartilage, subchondral bone sclerosis, pain, stiffness, and loss of function, imposing substantial socioeconomic and quality-of-life burdens [2]. Inflammation is a key contributor to OA progression, driving cartilage degradation and joint dysfunction [3]. Beyond interleukin-1β (IL-1 β), a variety of pro-inflammatory cytokines—including tumor necrosis factor α (TNF-α), interleukin 6 (IL-6), and chemokines such as CCL2 and CXCL8—play critical roles in orchestrating the inflammatory microenvironment in OA [4]. Concurrently, growth factors such as transforming growth factor β and vascular endothelial growth factor contribute to aberrant tissue remodeling and pathological angiogenesis within subchondral bone and the synovium, which can exacerbate inflammation, cartilage degradation, and pain [5]. Although considerable progress has been made in delineating these molecular mediators, the precise pathological mechanisms underlying OA remain incompletely understood, underscoring the multifactorial and heterogeneous nature of the disease. IL-1β activates the nuclear factor-kappa B (NF-κB) and mitogen-activated protein kinase (MAPK) signaling pathways. This signaling cascade upregulates pro-inflammatory cytokines, including TNF-α and IL-6, as well as inflammatory mediators such as nitric oxide (NO), and it induces matrix-degrading enzymes, including matrix metalloproteinase-9 (MMP-9) and MMP-13. These processes result in accelerated extracellular matrix (ECM) depletion, hyaluronan fragmentation, chondrocyte senescence, apoptosis, and, ultimately, cartilage breakdown [6]. Current analgesics and non-steroidal anti-inflammatory drugs relieve pain, but neither stops cartilage loss nor prevents long-term disability. Their systemic adverse effects further highlight the need for safer disease-modifying interventions. In this context, natural plant-derived products have garnered attention as potential sources of novel therapies due to their multi-target bioactive compounds, relatively low toxicity, and long-standing traditional use. Such interventions could therefore complement existing therapies by modulating inflammatory pathways, attenuating cartilage degradation, and preserving joint homeostasis.

*Elaeagnus angustifolia* L. is commonly known as oleaster or Russian olive [7]. It has long been used in traditional medicine, particularly the therapeutic applications of its fruits, which are recognized for their tonic, nutritive, antiulcerogenic, and antipyretic properties [8]. In addition, preclinical studies in animal models of OA and inflammation have shown that oleaster extract decreases MMP-13 levels, reduces inflammatory cell infiltration, and alleviates pain [9,10]. Clinical studies have demonstrated that oral administration of oleaster extract (300–600 mg/day) significantly reduces pain (VAS, LPFI, and PGA). These studies also reported improved function (WOMAC scores), with efficacy comparable to ibuprofen [11,12]. Building on these traditional and clinical uses, recent pharmacological studies have further validated these traditional uses by revealing that oleasters possess significant wound-healing, antibacterial, and anti-inflammatory properties [13,14]. Phytochemical investigations of oleaster fruit extracts have revealed a diverse array of bioactive constituents, including flavonoids, polysaccharides, sitosterols, cardiac glycosides, terpenoids, coumarins, phenolic acids, amino acids, saponins, carotenoids, vitamins, and tannins [8]. A recent LC-MS-based phytochemical investigation of the *E. angustifolia* fruit identified 56 distinct constituents, 26 of which demonstrated in vitro anti-inflammatory activity. Notably, triterpenes, phenolic compounds, and organic acids have been identified as key contributors to these anti-inflammatory effects [15].

*Sophora japonica* L. (*Styphnolobium japonicum*), a perennial woody species of the legume family, is an edible medicinal plant with a long history of traditional use and modern pharmacological interest. Native to China, and subsequently disseminated across various Asian and European regions through cultivation, *S. japonica* has been used for over two millennia in traditional medicine, particularly for inflammatory conditions, blood circulation improvement, musculoskeletal disorders, heat clearance, detoxification, blood pressure regulation, and hemostatic purposes [16]. Preclinical studies on *S. japonica* fruit extract have demonstrated its anti-inflammatory and bone-protective effects across multiple models. In vitro, the extract reduced pro-inflammatory mediators (NO, PGE2, IL-6, and IL-1β) via the inhibition of NF-κB and MAPK signaling in LPS-stimulated RAW 264.7 macrophages and promoted osteogenic growth factors (IGF-I and TGF-β) in rat-derived osteoclast precursor cells [17,18]. In vivo, the extract improved bone mineral density, modulated bone turnover markers, and reduced inflammatory cytokine levels in ovariectomized rabbits and rats as well as in collagen-induced arthritic mice [19,20,21]. Among the various parts, including the flowers, fruits, bark, branches, and leaves of *S. japonica* used for medicinal purposes, the fruits are particularly rich in the diverse phytochemicals that substantially contribute to the pharmacological profile of the plant [22]. Notably, the fruits contain an abundance of flavonoids, such as quercetin, kaempferol, and rutin and a variety of complex kaempferol glycosides, which are believed to play key roles in exerting antioxidant, anti-inflammatory, and vascular protective effects [23].

Recently, the concept of synergy has garnered increasing attention in phytotherapy [24,25]. Synergy refers to the phenomenon in which the combined therapeutic effects of multiple plant extracts exceed the effects of individual extracts [26]. Combining herbal extracts with diverse phytochemical profiles, such as flavonoids, phenolics, and terpenoids, can enhance anti-inflammatory, antioxidant, and anti-photoaging activities [27]. For example, combinations of *Panax ginseng* with *Salvia miltiorrhiza*, *Artemisia annua*, *Gardenia jasminoides*, and *Rheum palmatum* have shown greater efficacy than single extracts in cardiovascular or liver injury models [28,29]. However, despite the traditional, clinical, and preclinical evidence supporting their joint health benefits, no studies have explored the synergistic effects of these two fruit extracts in the context of joint inflammation or cartilage degradation. Therefore, this study aimed to evaluate the synergistic protective effects of oleaster fruit and *S. japonica* L. fruit extracts against IL-1β-induced inflammation in human chondrocytes.

## 2. Materials and Methods

### 2.1. Materials

Dimethyl sulfoxide (DMSO), 3-(4,5-dimethylthiazol-2-yl)-2,5-diphenyltetrazolium bromide (MTT), and quercetin were purchased from Sigma–Aldrich (St. Louis, MO, USA). The primary human chondrocytes and corresponding growth media were obtained from PromoCell GmbH (Heidelberg, Germany). All other chemicals and reagents used were of analytical grade, unless otherwise specified.

### 2.2. Preparation of Oleaster Fruit Extract (OE), S. japonica L. Fruit Extract (SJE), and Their Mixtures

OE, SJE, and their mixtures were supplied by NOVAWells Co. Ltd. (Cheongju, Republic of Korea). For OE, whole fruits were extracted using 50% (*v*/*v*) ethanol at 70 ± 5 °C for 4 ± 1 h, with a solvent-to-solid ratio of 10:1 (*w*/*v*). The resulting crude extract was filtered through a polyethylene cartridge filter, concentrated under reduced pressure by rotary evaporation, and freeze-dried to remove residual moisture. SJE was imported from GreenChem (Bengaluru, India; lot no. REX/24016) by NOVAWells Co. Ltd. It was prepared by extracting the fruits with 60% (*v*/*v*) ethanol at 80 ± 5 °C for 4 ± 1 h. The extract was concentrated under vacuum, cooled, and subjected to low-temperature precipitation for 16–24 h. Approximately one-third of the supernatant was decanted, and the remaining concentrate was spray-dried to obtain a powdered extract. SJE was standardized to contain more than 10% sophoricosides. To prepare the OE–SJE mixtures, the oleaster concentrate was blended with SJE at solid content ratios of 3:1, 2:1, 1:1, 1:2, and 1:3 (*w*/*w*), followed by freeze-drying to yield the final powdered complexes.

### 2.3. Cell Culture and Protective Effect

Human chondrocytes (passage 3–5) were grown in complete chondrocyte growth medium supplemented with 10% fetal calf serum at 37 °C in a humidified atmosphere containing 5% CO_2_. The cells were seeded into 96-well plates at a density of 1.0 × 10^4^ cells/mL and allowed to attach for 24 h. After attachment, the serum-free mediums containing the samples (25–100 µg/mL) were treated for 24 h, with or without IL-1β (10 ng/mL). Following treatment, cell viability (cytotoxicity and protective effect) was assessed using the MTT assay. Specifically, 20 µL of MTT reagent (5 mg/mL) was added to each well and incubated for 2 h. The medium was then removed, and DMSO was added to dissolve the formazan crystals. For the measurement of cytokines, NO, MMPs, and hyaluronan levels, the culture supernatants were centrifuged to remove cells and debris, and the clear supernatants were collected.

### 2.4. Measurement of TNF-α, IL-6, and NO Levels

TNF-α (KHC3011) and IL-6 (KHC0061) ELISA kits (Invitrogen, Carlsbad, CA, USA) were used to detect and quantify the cytokine levels in the culture supernatants, following the manufacturer’s instructions. NO production was measured using Griess reagent. Cell culture supernatants (100 µL) were mixed with 100 µL of Griess reagent in 96-well plates (SPL lifesciences, Pocheon, Republic of Korea) and incubated for 10 min in the dark at room temperature (18–25 °C). Absorbance was measured at 540 nm using a microplate reader (Bio-Tek, Inc., Winooski, VT, USA). The concentration of nitrite, which is a stable NO metabolite, was calculated using a sodium nitrite standard curve.

### 2.5. Measurement of MMP-9, MMP-13, and Hyaluronan

The levels of MMP-9 and MMP-13 were quantified using the human MMP-9 and human MMP-13 ELISA kits (Invitrogen). Hyaluronan concentration was measured using the Hyaluronan DuoSet ELISA kit (R&D Systems, Minneapolis, MN, USA). All ELISAs were performed according to the manufacturers’ instructions.

### 2.6. Evaluation of Synergistic Effect

The synergistic effects were assessed using the combination index (CI) model described by Chou [30]. The interaction of combinations was calculated using ‘‘CompuSyn’’ software ver 1.0 (CompuSyn, Inc., Paramus, NJ, USA), and CI was determined using the following isobologram equation:

CI = d_1_/D_1_ + d_2_/D_2,_

where D_1_ and D_2_ are the concentrations of OE and SJE, respectively, which are required individually to produce a chosen effect level, and d_1_ and d_2_ are the concentrations of OE and SJE within the mixture required to achieve the same effect. For example, in the 3:1 combination at a total concentration of 100 µg/mL, d_1_ = 75 µg/mL (OE) and d_2_ = 25 µg/mL (SJE). CI was calculated using all tested concentrations of 25, 50, and 100 µg/mL for OE, SJE, and the 3:1 combination. CI values of < 1 indicate a synergistic effect.

### 2.7. Statistical Analysis

All experiments were performed at least three times. The data are presented as the means ± standard errors. Statistical analyses were conducted using GraphPad Prism version 8.2.1 (GraphPad Software, San Diego, CA, USA). One-way analysis of variance was used to assess the differences between the groups, followed by Tukey’s post-hoc test for multiple comparisons. Statistical significance was set at *p* < 0.05.

## 3. Results and Discussions

### 3.1. Protective Effects of OE, SJE, and the OE:SJE Combinations Against IL-1β-Induced Inflammation in Human Chondrocytes

**Figure 1:**
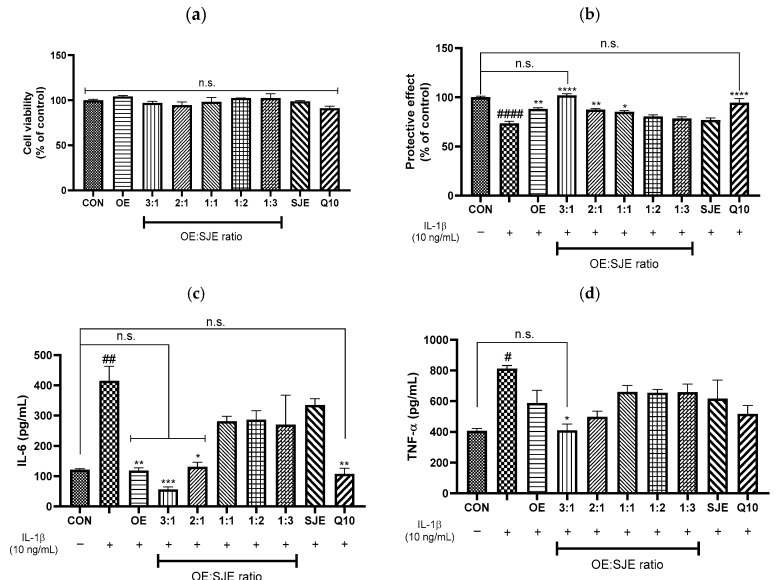
(**a**) Cell cytotoxicity, (**b**) protective effect, (**c**) IL-6 levels, and (**d**) TNF-α levels in human chondrocytes. Quercetin 10 µM (Q10) was used as a positive control. OE and SJE were each used at 100 µg/mL for the single treatments. The combination treatments (OE:SJE) were applied at ratios of 3:1, 2:1, 1:1, 1:2, and 1:3, with a total concentration of 100 µg/mL. Each value is expressed as a mean ± standard error (*n* = 3). ^#^ *p* < 0.05, ^##^ *p* < 0.01, and ^####^ *p* < 0.0001 versus the control cells; * *p* < 0.05, ** *p* < 0.01, *** *p* < 0.001, and **** *p* < 0.0001 versus the IL-1β-treated group. CON, control group; OE, oleaster fruit extract; SJE, *Sophora japonica* L. fruit extract; n.s., not significant.

In the present study, we evaluated the protective effects of various combinations of OE and SJE against IL-1β-induced inflammatory injury in human chondrocytes. A range of OE:SJE ratios (3:1, 2:1, 1:1, 1:2, and 1:3) was tested at a concentration of 100 µg/mL. This concentration (100 µg/mL) was selected based on preliminary observations indicating that OE exhibited cytotoxic effects in human chondrocytes at concentrations above 125 µg/mL; therefore, 100 µg/mL was determined to be the highest non-toxic concentration suitable for the subsequent experiments. The MTT assay confirmed that none of the mixtures exhibited cytotoxicity (Figure 1a). While IL-1β stimulation significantly reduced chondrocyte viability (*p* < 0.0001), treatment with the OE and 3:1, 2:1, and 1:1 OE:SJE mixtures significantly restored cell viability (Figure 1b). Importantly, only the 3:1 combination showed no significant difference from the control group, indicating full restoration of cell viability to control levels. The OE, 2:1 combination, and 1:1 combination treatments increased viability to levels close to that of the control group, suggesting a partial but meaningful restoration. Furthermore, inflammatory cytokine analysis revealed that IL-6 and TNF-α levels were markedly reduced by the 3:1 combination (Figure 1c,d) compared to IL-1β-treated group. In addition, the 3:1 combination restored IL-6 and TNF-α levels comparable to those in the control group. A previous study reported that SJE significantly suppressed pro-inflammatory cytokine production in lipopolysaccharide-stimulated RAW264.7 macrophages, with the most pronounced effects observed at 500 µg/mL [17]. Notably, IL-6 levels were not significantly reduced at 100 µg/mL, a finding consistent with our current results despite the differences in cell types used. Additionally, S. japonica alleviates collagen-induced arthritis by downregulating pro-inflammatory cytokines and mediators, including NF-κB signaling, and suppressing osteoclastic bone remodeling in joint tissues [20]. Oleaster exhibits strong antioxidant and anti-inflammatory activities. Its bioactive compounds, particularly flavonoids and phenolic compounds, protect chondrocytes from oxidative damage and reduce inflammation. Traditional use and recent studies support its analgesic and anti-inflammatory effects, which are partly attributed to the inhibition of cyclooxygenase enzymes and the suppression of inflammatory cytokine production, suggesting its potential as a natural therapeutic agent for OA [8]. Collectively, the findings suggest that the 3:1 OE:SJE combination may exert synergistic protective effects against IL-1β-induced inflammation in human chondrocytes and can restore cell viability and inflammatory cytokine levels to those comparable to the control group.

### 3.2. Synergistic Effect of the 3:1 OE:SJE Combination Against IL-1β-Induced Inflammation in Human Chondrocytes

**Figure 2:**
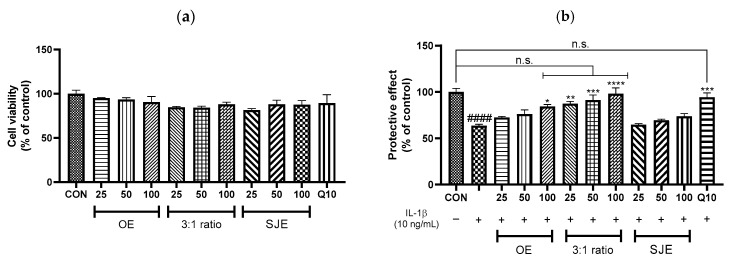
Effects of OE, SJE, and the 3:1 OE-SJE combination on (**a**) cell cytotoxicity and (**b**) protection. Each value is expressed as the mean ± standard error (*n* = 3). ^####^ *p* < 0.0001 versus the control cells; * *p* < 0.05, ** *p* < 0.01, *** *p* < 0.001, and **** *p* < 0.0001 versus the IL-1β-treated group. CON, control group; OE, oleaster fruit extract; SJE, *Sophora japonica* L. fruit extract; n.s., not significant.

In pharmacology, synergy refers to an interaction in which the combined effect of two agents exceeds the expected outcome based on their individual effects [30]. This contrasts with additive effects, in which the overall response is simply the arithmetic sum of the contribution of each agent. Synergistic interactions often involve cooperative or complementary mechanisms that amplify biological outcomes beyond simple summation [31]. Quantitative analyses using validated models, such as the median-effect method and CI analysis, are essential for accurately distinguishing between additive and synergistic interactions. To further explore the potential synergistic effects of OE and SJE, we assessed their anti-inflammatory activity at concentrations of 25, 50, and 100 µg/mL and evaluated synergism based on CI values. None of the treatments were cytotoxic at any of the tested concentrations (Figure 2a). IL-1β stimulation significantly reduced cell viability by 36.5% compared with the control group. Treatment with OE, SJE, or their 3:1 combination led to a dose-dependent recovery of cell viability. Notably, the 3:1 combination at 100 µg/mL restored cell viability by 54.6% compared with that of IL-1β-treated cells, demonstrating the highest protective efficacy among all tested conditions. Furthermore, the protective effect of the 3:1 combination at 100 µg/mL was comparable to that of quercetin (10 µM), a well-known flavonoid with anti-inflammatory properties (Figure 2b). The 3:1 OE:SJE combination restored viability to levels not significantly different from the control group at all tested concentrations (25, 50, and 100 µg/mL), indicating a consistent and robust protective effect across the entire dose range.

**Figure 3:**
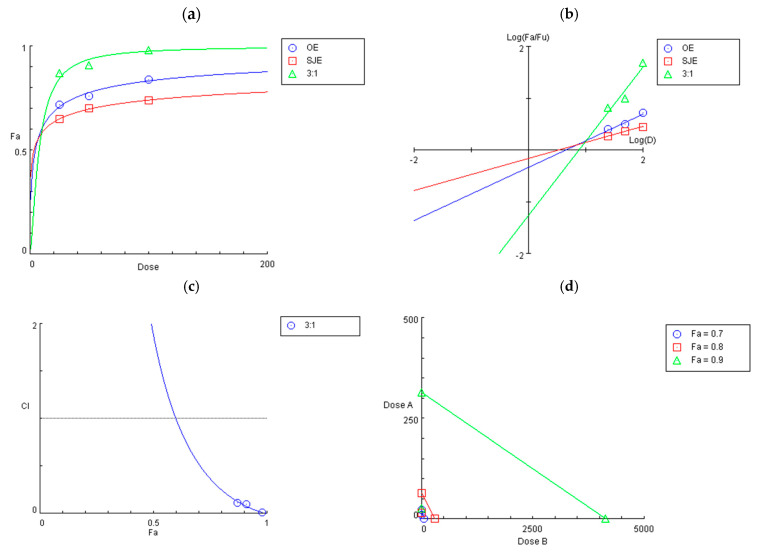
(**a**) Dose-effect curves of OE, SJE, and the 3:1 OE-SJE combination in human chondrocyte viability. (**b**) Median-effect plot of OE, SJE, and the 3:1 OE-SJE combination. (**c**) Combination index (CI) plot. (**d**) Isobologram curves of OE, SJE, and the 3:1 OE-SJE combination. OE, oleaster fruit extract; SJE, *Sophora japonica* L. fruit extract; Fa, fraction affected; Fu, fraction unaffected; D, dose; dose A, dose of OE; dose B, dose of SJE.

In the present study, the OE and SJE combination exhibited a strong synergistic effect in protecting chondrocytes from IL-1β-induced inflammatory stress. As shown in the dose-effect and median-effect plots (Figure 3a,b), the combination treatment shifted the response curve upward and to the left, suggesting enhanced potency compared to the single treatments. According to the Chou–Talalay method [30], the calculated CI values for the 3:1 OE:SJE mixture were 0.52, 0.25, and 0.09 at 70%, 80%, and 90% cell viability, respectively (Figure 3c). All values were well below 1.0, indicating a strong synergistic interaction across multiple effect levels. These results also indicate a substantial reduction in the dose required to achieve the desired effect. For instance, achieving 90% cell viability required 313.6 µg/mL of OE or 4135.8 µg/mL of SJE individually, whereas the combination required only 26.4 µg/mL of OE and 8.8 µg/mL of SJE, highlighting a marked dose reduction (Figure 3d). Previous studies have suggested five possible mechanisms underlying the synergistic effects of phytochemical combinations: (1) enhanced bioavailability, (2) increased antioxidant capacity, (3) interactions with the gut microbiome, (4) targeting the same signaling pathways, and (5) targeting different but complementary signaling pathways [32]. Among these, the contributions from enhanced bioavailability and gut microbiome-mediated interactions were inherently excluded from the present in vitro cellular model. Therefore, the synergistic effect observed with the 3:1 OE:SJE combination was most likely mediated by the enhanced antioxidant capacity and modulation of anti-inflammatory signaling pathways, involving both convergent and complementary molecular targets.

### 3.3. Inhibitory Effects of the 3:1 OE:SJE Combination on Inflammatory and Cartilage Matrix Degradation Markers in IL-1β-Induced Human Chondrocytes

**Figure 4:**
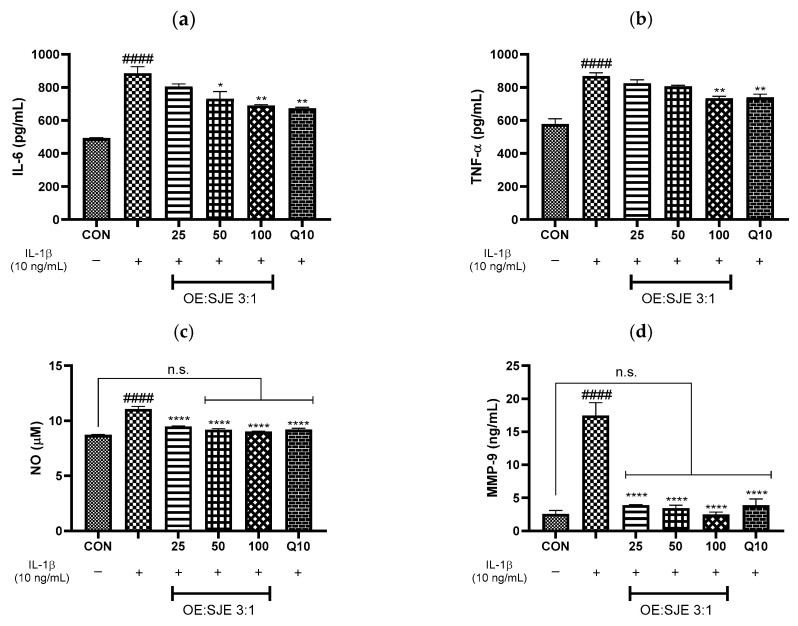

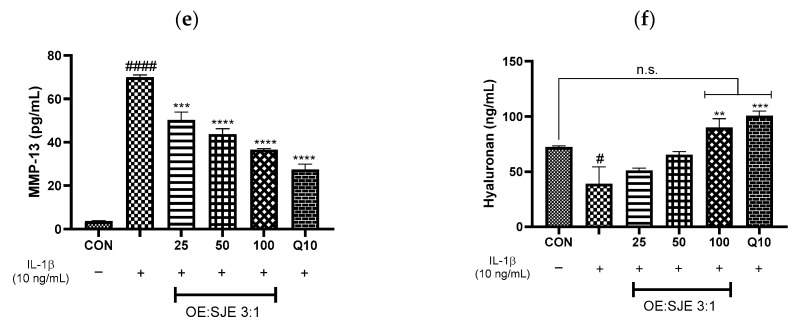
Effects of OE, SJE, and the 3:1 OE-SJE combination on (**a**) IL-6, (**b**) TNF-α, (**c**) NO, (**d**) MMP-9, (**e**) MMP-13, and (**f**) hyaluronan levels in human chondrocytes. Each value is expressed as a mean ± standard error (*n* = 3). ^#^ *p* < 0.05 and ^####^ *p* < 0.0001 versus the control cells; * *p* < 0.05, ** *p* < 0.01, *** *p* < 0.001, and **** *p* < 0.0001 versus the IL-1β-treated group. CON, control group; OE, oleaster fruit extract; SJE, *Sophora japonica* L. fruit extract; n.s., not significant.

OA progression is characterized by a sustained inflammatory microenvironment marked by elevated levels of pro-inflammatory cytokines and increased activity of the matrix-degrading enzymes that drive ECM degradation and disrupt cartilage homeostasis [33]. Among these enzymes, MMP-13 is recognized as the primary collagenase responsible for degrading type II collagen, the major structural component of articular cartilage, thereby playing a central role in OA pathogenesis [34]. In contrast, MMP-9 preferentially degrades denatured collagen and other noncollagenous ECM constituents, facilitating subsequent matrix breakdown. The concerted action of MMP-13 and MMP-9 accelerates cartilage degradation and contributes to the progression of arthritic joint damage. Additionally, hyaluronan, a critical component of the cartilage matrix and synovial fluid, supports joint lubrication and tissue repair, and its depletion exacerbates joint dysfunctions. In the present study, IL-1β stimulation significantly increased the expression of pro-inflammatory mediators, IL-6, TNF-α, and NO, as well as the catabolic enzymes MMP-9 and MMP-13, while concomitantly reducing hyaluronan production in human chondrocytes, recapitulating the inflammatory and degradative milieu characteristic of OA joints. Treatment with the 3:1 OE:SJE combination dose-dependently attenuated these inflammatory mediators and matrix-degrading enzymes and restored hyaluronan levels, demonstrating potent anti-inflammatory and chondroprotective activities (Figure 4). Importantly, the 3:1 combination at 100 µg/mL not only significantly reduced IL-6, TNF-α, NO, MMP-9, and MMP-13 and increased hyaluronan compared with the IL-1β group but also restored NO, MMP-9, and hyaluronan levels to the control values. The marked suppression of IL-6, TNF-α, NO, MMP-9, and MMP-13, along with the restoration of hyaluronan levels, highlights the multifaceted chondroprotective efficacy of the 3:1 OE:SJE combination. Although many conventional OA treatments primarily address symptom relief, often targeting a single inflammatory mediator or enzymatic pathway, the present findings suggest that this phytochemical combination exerts a broader spectrum of action by concurrently modulating key cytokines, catabolic enzymes, and ECM components critical to OA pathogenesis. Although the present study did not involve the chemical profiling of OE and SJE, previous studies have provided important insights into their bioactive constituents and potential mechanisms [16,35,36]. OE is rich in flavonoids, such as quercetin, kaempferol, and phenolic acids, which inhibit MAPK and NF-κB activation and downregulate COX-2 expression in inflammatory models [32,37,38]. Similarly, SJE contains rutin, quercetin, genistein, and other isoflavones that are known to suppress pro-inflammatory cytokine expression by modulating the MAPK and JAK/STAT signaling pathways [17,22,35,39]. These compounds have also been reported to reduce MMP production and oxidative stress in joint tissues [40,41,42]. Taken together, although the exact molecular contributors to the observed synergistic effects were not directly identified in this study, the phytochemicals commonly found in OE and SJE may synergistically contribute to the suppression of IL-1β-induced inflammation and matrix degradation through overlapping and complementary signaling pathways.

It should be noted that the present study had certain limitations. An HPLC analysis confirmed the presence of the marker compounds quercetin-3-glucosyl-(1 → 2)-galactoside (Q-3-GG) in the OE and sophoricoside in the SJE (Figure S1). These markers may not fully represent the bioactive constituents responsible for the observed synergistic effects. Although Q-3-GG itself has not been directly demonstrated to exert anti-inflammatory or chondroprotective activity, previous studies have indicated that quercetin glycosides can undergo enzymatic hydrolysis in vivo, releasing quercetin aglycone with diverse biological activities [37,43,44]. Consistent with this, our positive control experiments using quercetin supported the potential contribution of the released aglycone to the observed effects. In addition, sophoricoside has been reported to exhibit anti-inflammatory activity in preclinical models [45]. Nevertheless, the current in vitro system does not fully replicate in vivo pharmacokinetic processes, including absorption, metabolism, distribution, and bioavailability. This raises uncertainty regarding which compounds, and in what forms or concentrations, actually reach chondrocytes following oral or topical administration. Therefore, while the present findings provide mechanistic insight into potential synergistic effects, their translational significance requires further validation.

## 4. Conclusions

This study demonstrated that the 3:1 OE:SJE combination exhibits strong synergistic protective effects against IL-1β-induced inflammation in human chondrocytes by enhancing cell viability, suppressing inflammatory mediators, and reducing matrix-degrading enzymes. Future studies should focus on the phytochemical analysis and mechanistic evaluation of individual components to elucidate the precise interactions driving the synergistic effects, thereby advancing the development of targeted therapeutics for OA.

## Data Availability

The original contributions presented in this study are included in the article or Supplementary Material. Further inquiries can be directed to the corresponding authors.

## Supplementary Materials

The following supporting information can be downloaded at: https://www.mdpi.com/article/10.3390/foods14173099/s1, Figure S1: HPLC chromatograms of the marker compounds. (A), quercetin-3-glucosyl-(1->2)-galactoside standard; (B), oleaster fruit extract (OE); (C), OE:SJE 3:1 combination; (D), sophoricoside standard; (E), *Sophora japonica* L. fruit extracts (SJE); (F), OE:SJE 3:1 combination.
