# Supplementary material for: Synergistic Protective Effects of Oleaster Fruit and Sophora japonica L. Fruit Extracts Against IL-1β-Induced Inflammation in Human Chondrocytes
Source: Foods. 2025 Sep 4;14(17):3099. doi: 10.3390/foods14173099 (PMC12428578; doi:10.3390/foods14173099)
Supplement: Supplementary file 1 [file foods-14-03099-s001.zip › foods-3821550-supplementary.pdf]

Supplementary Materials

Synergistic protective effects of oleaster fruit and *Sophora japonica* L. fruit extracts against IL-1β-induced inflammation in human chondrocytes

Hana Lee [1], Jinyeong Lim [2], Myeonghwan Oh [2,*], and Junsoo Lee [1,*]

[1] Department of Food Science and Biotechnology, Chungbuk National University, Cheongju, Chungbuk, 28644, Republic of Korea; dlgksk0514@naver.com (H. Lee); junsoo@chungbuk.ac.kr (J. Lee)

[2] Life Science Research Institute, NOVAWells Co., Ltd., 35 Yangcheongtaekji 3-gil, Ochang-eup, Cheongwon-gu, Cheongju, Chungbuk 28126, Republic of Korea; jinyglim@novarex.co.kr (J. Lim); omh4860@novarex.co.kr (M. Oh)

* Correspondence: omh4860@novarex.co.kr (M. Oh); junsoo@chungbuk.ac.kr (J. Lee)

Materials and Methods

Qualitative Analysis of Marker Compounds by HPLC

For the qualitative analysis of marker compounds, high-performance liquid chromatography (HPLC) was performed on both oleaster and *Sophora japonica* L. fruit extracts.

For oleaster fruit extract, accurately weighed samples were dissolved in distilled water and sonicated for 30 minutes. The standard compound, Quercetin-3-glucosyl-(1→2)-galactoside (ChemFaces, CFN95649, ≥98%), was dissolved in methanol. HPLC analysis was carried out with an injection volume of 5 μL, UV detection at 254 nm, a column temperature of 45 °C, and a flow rate of 1 mL/min. The mobile phase consisted of 0.05% formic acid in distilled water (Solvent A) and 0.05% formic acid in acetonitrile (Solvent B). The gradient program was as follows: 90% A and 10% B at 0–10 min, 83% A and 17% B at 15–20 min, and 90% A and 10% B at 25 min.

For *Sophora japonica* L. fruit extract, accurately weighed samples were dissolved in methanol and sonicated for 30 minutes. The standard compound, Sophoricoside (ChromaDex, ASB-00019324-025, ≥90%), was dissolved in methanol. HPLC conditions included an injection volume of 5 μL, UV detection at 260 nm, a column temperature of 30 °C, and a flow rate of 1 mL/min. The mobile phase consisted of 0.1% formic acid in distilled water (Solvent A) and 0.1% formic acid in acetonitrile (Solvent B). The gradient program was as follows: 100% A and 0% B at 0–10 min, 90% A and 10% B at 15–20 min, 85% A and 15% B at 25–35 min, 82% A and 18% B at 40–50 min, 0% A and 100% B at 55–60 min, and 100% A and 0% B at 65–70 min.

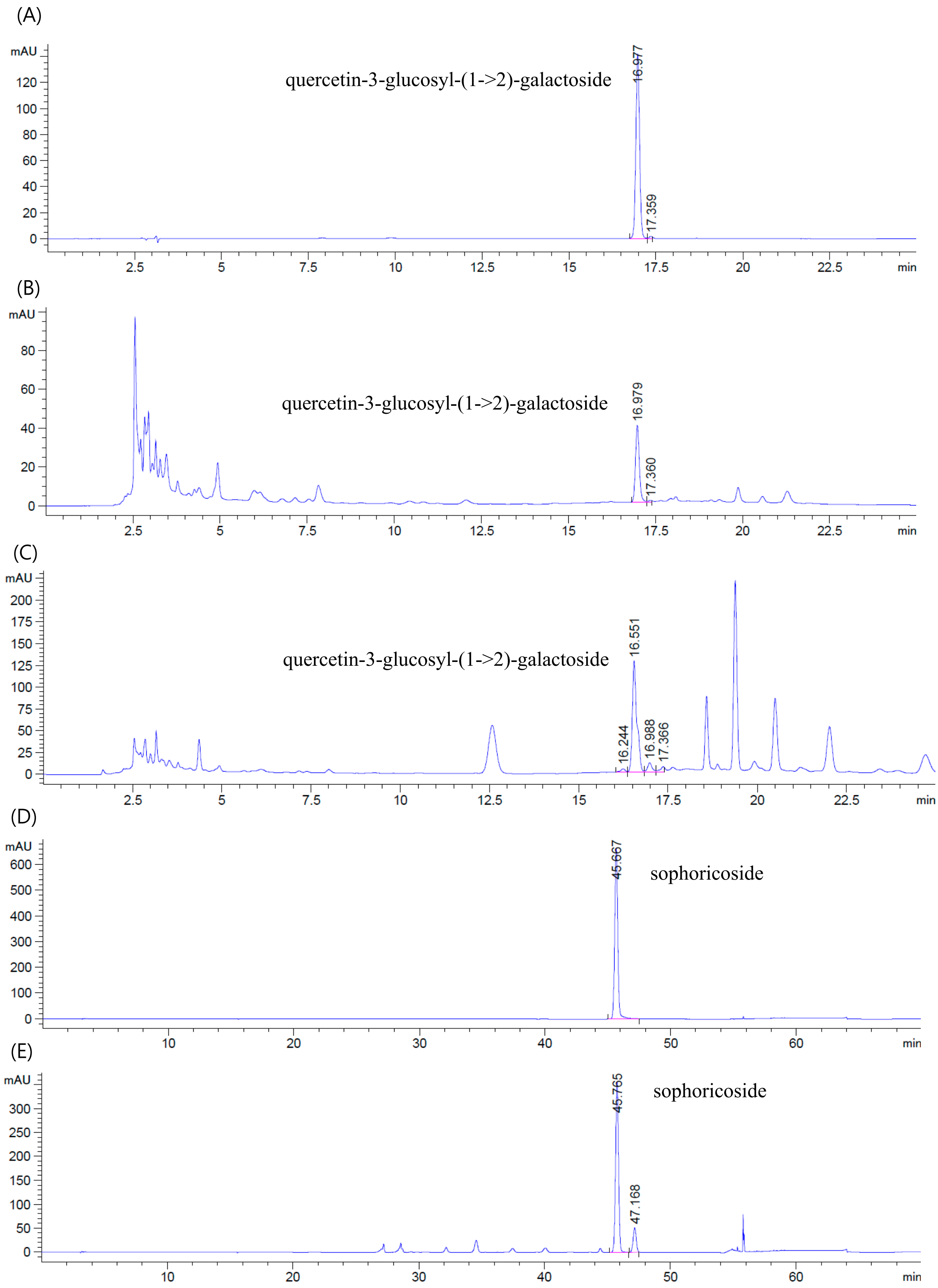

(A)
mAU
min
quercetin-3-glucosyl-(1->2)-galactoside
16.977
17.359
(B)
mAU
min
quercetin-3-glucosyl-(1->2)-galactoside
16.979
17.360
(C)
mAU
min
quercetin-3-glucosyl-(1->2)-galactoside
16.551
16.244
16.988
17.366
(D)
mAU
min
sophoricoside
45.667
(E)
mAU
min
sophoricoside
45.765
47.168

(F)

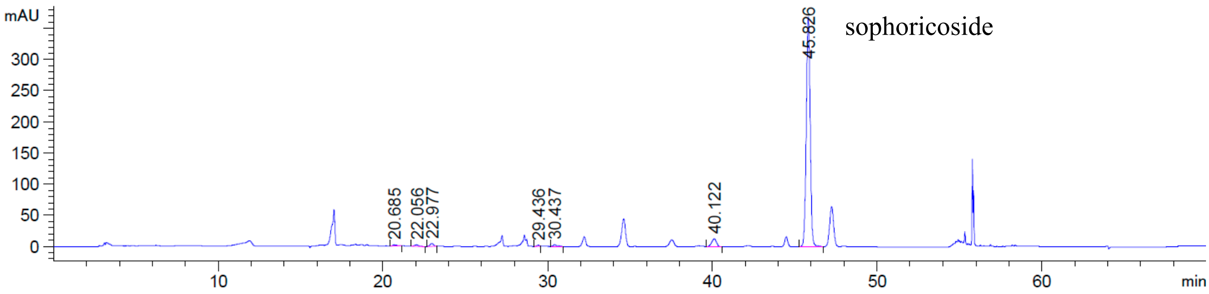

Figure S1. HPLC chromatograms of the marker compounds. (A), quercetin-3-glucosyl-(1->2)-galactoside standard; (B), oleaster fruit extract (OE); (C), OE:SJE 3:1 combination; (D), sophoricoside standard; (E), *Sophora japonica* L. fruit extracts (SJE); (F), OE:SJE 3:1 combination.
